# Pancreaticoduodenectomy preserving aberrant gastroduodenal artery utilized in a previous coronary artery bypass grafting: A case report and review of literature

**DOI:** 10.1097/MD.0000000000027788

**Published:** 2021-12-03

**Authors:** Takeo Toda, Hideyuki Kanemoto, Satoshi Tokuda, Akihiko Takagi, Noriyuki Oba

**Affiliations:** a Department of Gastroenterological Surgery, Shizuoka General Hospital, 4-27-1 Kita-Ando, Aoi-Ku, Shizuoka-Shi, Shizuoka, Japan; b Department of Gastroenterological Surgery, Shizuoka General Hospital, 4-27-1 Kita-Ando, Aoi-Ku, Shizuoka-Shi, Shizuoka, Japan.

**Keywords:** computed tomography angiography, coronary artery bypass grafting, pancreaticoduodenectomy, replaced gastroduodenal artery, superior mesenteric artery

## Abstract

**Rationale:**

Pancreaticoduodenectomy (PD) is a technically demanding procedure with high rates of morbidity and mortality. Therefore, preoperative evaluation of anatomy is indispensable. Multi-detector row computed tomography (CT) enables us to precisely understand arterial anatomy. It is a well-known fact that anatomical variants are often present in the hepatic artery (HA) but rarely in the gastroduodenal artery (GDA). We present the case of a patient with ampullary cancer with a rare anatomical anomaly, “replaced GDA (rGDA) ” arising from the superior mesenteric artery, along with a history of coronary artery bypass grafting (CABG) using right gastroepiploic artery (RGEA).

**Patient concerns:**

A 69-year-old male patient was referred to our department for further investigation of elevated hepatobiliary enzymes. He presented with no symptoms besides intermittent fever of 38°C. He had an operative history of CABG using the RGEA.

**Diagnosis:**

Abdominal CT and esophagogastroduodenoscopy showed an ampullary tumor and biopsy specimen from the lesion revealed adenocarcinoma. CT angiography revealed the rGDA instead of a normal common HA.

**Intervention:**

We performed a safe PD, preserving the rGDA and the RGEA to maintain hepatic and cardiac perfusion.

**Outcomes:**

Owing to the presence of a refractory pancreatic fistula, the length-of-hospital stay was extended, and he was discharged on postoperative day 72 without vascular complications. At present, the patient is in good physical condition and does not present with cardiovascular complications as well as tumor recurrence at 6 months after surgery.

**Lessons:**

This is possibly the first case of a patient who underwent PD and has a proper HA following a GDA arising from a superior mesenteric artery (rGDA) and has a previous operative history of CABG using the gastroepiploic artery. The coexistence of the history of cardiovascular surgery made PD for this patient considerably more challenging.

In the case of a rare anatomical anomaly, a coronary artery bypass via the RGEA should not be considered as an obstacle when R0 resection is achievable.

## 1 Introduction

Pancreaticoduodenectomy (PD) is a technically demanding procedure with high rates of morbidity and mortality.^[1]^ Hence, anatomical knowledge is essential before surgery.^[2]^ Identifying arterial abnormalities is the key to reduce ischemic or hemorrhagic morbidity and mortality.^[3]^ Multi detector-row computed tomography (MDCT) and CT angiography (CTA) enable us to understand the anatomical structure of blood vessels.^[4–6]^ It is well known that the hepatic artery (HA) has several anatomical variants.^[7,8]^ Contrarily, a gastroduodenal artery (GDA) variation is scarce and causes concern.^[9]^ GDA usually arises from the common HA (CHA) as a branch of the celiac axis (CeA), and the vessel is usually dissected during conventional PD. We experienced the case of ampullary cancer with a rare arterial anomaly, replaced GDA (rGDA),^[9–11]^ which originates from the superior mesenteric artery (SMA). The patient also had an operative history of coronary artery bypass grafting (CABG) using the right gastroepiploic artery (RGEA). Finally, both were safely preserved, and the patient was discharged without any cardiovascular event.

## 2 Case presentation

A 69-year-old male patient with elevated hepatobiliary enzymes was referred to our department for additional investigation. He presented with no symptoms but an intermittent fever of 38°C. The laboratory analysis provided the following results: serum aspartate aminotransferase, 100 U/L (normal range, 13–30 U/L); alanine aminotransferase, 224 U/L (normal range, 10–42 U/L); alkaline-phosphatase, 1400 U/L (normal range, 0–359 U/L); γ-glutamyl transpeptidase, 1061 U/L (normal range, 0–55 U/L); amylase, 12 U/L (normal range, 40–126 U/L); and total bilirubin, 1.2 mg/dL (normal range, 0.4–1.5 mg/dL); the serum level of CeA and CA19-9 were within the normal range.

**Figure 1:**
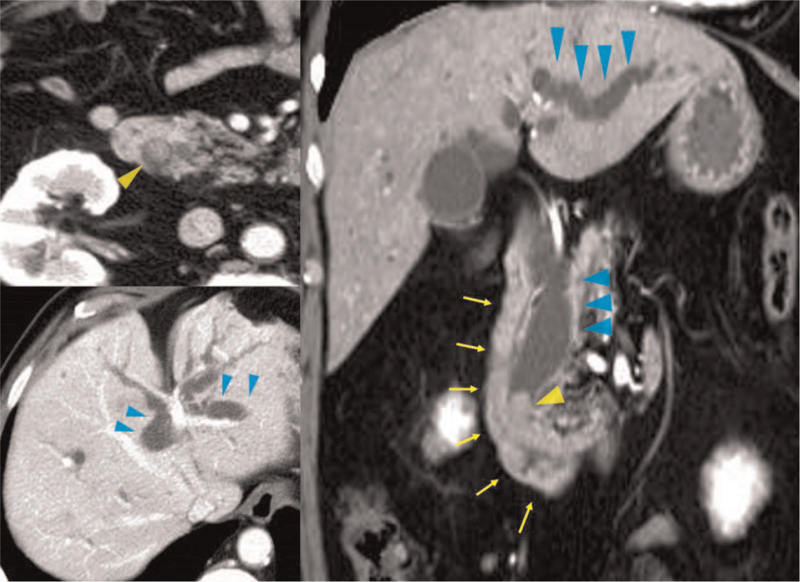
Contrast-enhanced abdominal CT revealed a 1 cm-sized tumor (yellow arrowhead) in the distal bile duct. The common bile duct and intrahepatic bile duct were dilated (blue arrowhead) due to this tumor. Wall thickening was seen in the second portion of the duodenum (yellow arrow). The endoscopic finding suggested the wall thickening as the mucosal lesion.

**Figure 2:**
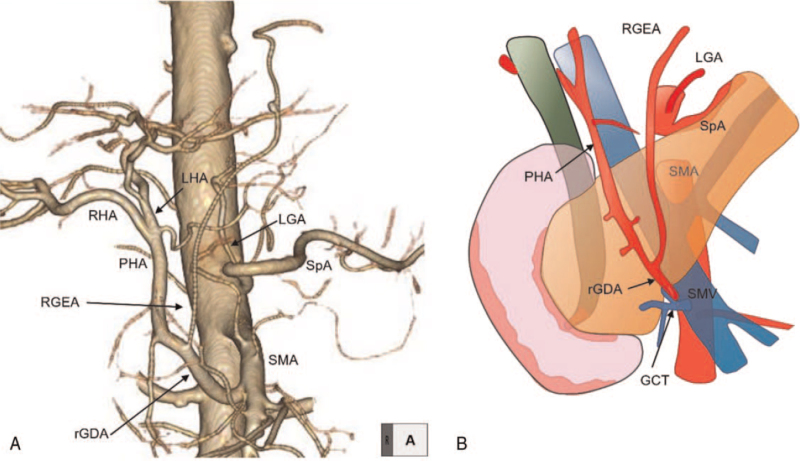
(A) 3D-CTA (B) Schema of this case. Imaging study revealed rGDA arising from the SMA, which ascended superior to the pancreatic parenchyma, and absence of CHA from the CeA. rGDA branched several arteries including the RGEA graft and terminated as a PHA. CeA = celiac axis, CHA = common hepatic artery, CTA = CT angiography, GCT = gastro-colic trunk, LGA = left gastric artery, LHA = left hepatic artery, PHA = proper hepatic artery, rGDA = replaced gastroduodenal artery, RGEA = right gastroepiploic artery, RHA = right hepatic artery, SMA = superior mesenteric artery, SMV = superior mesenteric vein, SpA = splenic artery.

Contrast-enhanced CT showed a tumor with a diameter of 10 mm around the ampulla of the duodenum (Fig. 1). The upstream common bile duct and intrahepatic bile duct expanded significantly but the pancreatic duct did not. Moreover, the wall thickening of the second part of the duodenum was visible. No distant metastases or lymph node metastases were noted. Esophagogastroduodenoscopy showed a tumor of exposed protruding type, and biopsy from the lesion revealed adenocarcinoma. Additionally, the thickening of the mucosa suggested adenoma as well. When evaluating vascular anatomy, 3D CTA revealed a rare vascular anomaly (Fig. 2A). CHA from the celiac artery was absent, and there was a proper hepatic artery (PHA) following GDA arising from the SMA instead. The artery ascended anterior to the surface of the pancreas and had branches corresponding to RGEA or superior pancreaticoduodenal artery, after which the GDA continued predominantly and ended with the PHA passing through the hepatoduodenal ligament. Furthermore, the patient had undergone CABG utilizing RGEA for angina pectoris when he was 50-year-old. By this procedure, RGEA was passing through the surface of the stomach and liver, eventually reached the mediastinum. Due to this operative history, he has a median sternotomy and laparotomy scar. No other findings were observed on physical examination.

**Figure 3:**
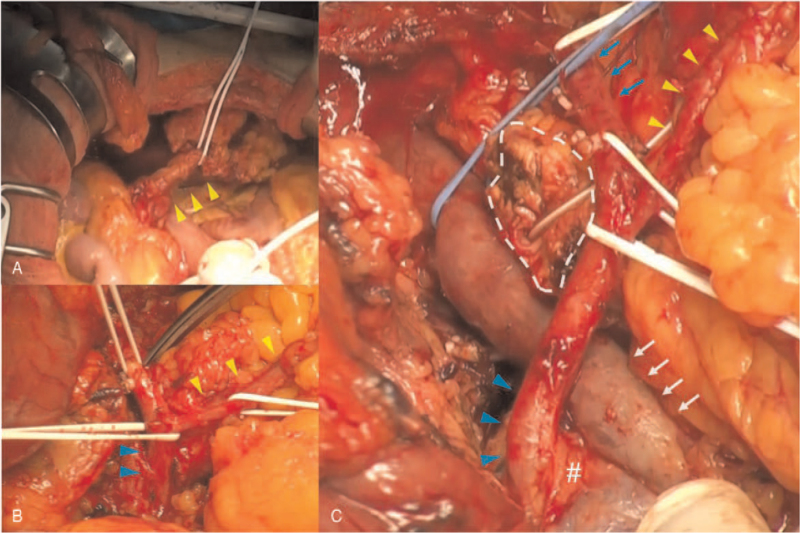
RGEA bypass graft (yellow arrowhead) was running through ante-gastric and ante-hepatic route and into the pericardiac space (A). Dissection of rGDA (blue arrowhead) preserving cardiac bypass graft and mobilized from pancreatic parenchyma (B). Pancreatic parenchyma was transected (surrounded by dashed white line) just above SMV (white arrow), and dissection of pancreatic nerve plexus had been performed (C). rGDA was derived from SMA (#) and terminated as proper hepatic artery (blue arrow). rGDA = replaced gastroduodenal artery, RGEA = right gastroepiploic artery, SMA = superior mesenteric artery, SMV = superior mesenteric vein.

Since the tumor demonstrated no invasion in the vascular system, we planned to perform conventional PD with total preservation of the rGDA to maintain cardiac and hepatic perfusion. The procedure began with an abdominal exploration through an upper to middle abdominal midline laparotomy, confirming the RGEA graft pulsating. The artery was found running anterior to the left lateral lobe of the liver into the pericardial space (Fig. 3A). Intraoperative findings showed no liver metastasis or disseminated nodules; it was decided that the operation would be continued. Dissection around the superior mesenteric vein revealed aberrant GDA on the anterior surface of the pancreatic neck. The artery emerged from a crevice between the lower border of the pancreatic neck and gastrocolic trunk. After the division of the stomach, we carefully dissected the pancreatic branches from the rGDA and preserved the RGEA to mobilize the rGDA totally from the pancreatic parenchyma (Fig. 3B). Next, lymph node dissection of the hepatoduodenal ligament and division of the distal duodenum was performed. Finally, the pancreas was transected above the superior mesenteric vein, and transection between SMA and pancreatic nerve plexus was performed in the usual method (Fig. 3C) to accomplish resection. Reconstruction was performed by the modified Child method, and during operation, we continuously monitored electrocardiography with no significant changes detected in the ST segment. Postoperatively, the hospital stay was extended because of the refractory pancreatic fistula. The pancreatic fistula was managed by persistent drainage and periodical tube exchange. We did not require additional drainage tubes after the operation. Postoperative CT revealed patent PHA and RGEA. He was discharged on the 72^nd^ postoperative day without cardiovascular complications. Histopathological revealed well differentiated adenocarcinoma classified as clinically T1 (localized to lamina propria mucosae) and N0, M0 (without lymphnode and distant metastasis) according to TNM Classification of Malignant Tumors, 8^th^ Edition. Drainage tube for pancreatic fistula was removed on the 105^th^ postoperative day at outpatient unit. At present, the patient is in good physical condition without cardiovascular complications and tumor recurrence at 6 months after surgery.

## 3 Discussion

In hepatobiliary-pancreatic neoplasm surgery, preoperative examination of anatomical abnormalities and tumor resectability is essential. MDCT and CTA play an important role in it.^[4]^ It is well known that HA typically has anatomical variants, and many studies have been accomplished to classify these variations.^[7,12–15]^ Meta-analysis on surgical, radiological, and cadaveric anatomical studies indicated a range of 11% to 45% in HA variation.^[7,8,16–20]^ The replaced right HA or the replaced CHA was in around 10% to 12% or 1% to 3%, respectively. These HA derived from SMA usually runs posteriorly and through the pancreatic nerve plexus, which requires special attention to preserve during PD.^[2,21]^ Contrarily, variation of GDA is scarce. According to Lipshutz^[12]^ report, GDA originates from CHA in 92% of the cases. The name “replaced GDA” refers to the artery which derived from SMA that passes through the front of the neck of the pancreas during its course. The artery branched superior pancreaticoduodenal artery and RGEA, and finally terminates in the PHA. Only a few available studies have mentioned rGDA,^[9]^ and there are only 3 reported cases of PD similar to the kind of anomaly in our case study^[10,11,22]^ (Table 1). The exception is that in one of these cases, the rGDA was terminated to the left HA, and the right HA was derived from the CeA. There was no mortality due to operation, but 1 patient had a postoperative myocardial infarction in the reported cases.

**Table 1:** Documents of pancreaticoduodenectomy in patient with replaced gastroduodenal artery.

Year	Author	Age	Gender	Malignancy	Operation	Termination of rGDA	Morbidity	Mortality
2017	Patil et al^[10]^	47	M	Pancreatic cancer	PD	PHA	unspecified	None
2017	CreTu et al^[22]^	60	M	Pancreatic cancer	PD	PHA	Myocardial infarction	None
2016	Younan et al^[11]^	55	F	Ampullary cancer	PD	LHA	None	None
2021	Our case	69	M	Ampullary cancer	PD	PHA	Pancreatic fistula	None

Preoperatively identifying rGDA prevents its misrecognition as a normal GDA, which descends on the surface of the pancreas during operation. Furthermore, it prevents ligation of the rGDA at the upper border of the pancreas when lymph node dissection of the hepatoduodenal ligament which is usually performed before transection between the pancreatic nerve plexus and SMA. The misidentification of rare vascular abnormalities such as ours can lead to serious complications, such as total hepatic ischemia, so the performance of PD requires precise anatomical understanding using MDCT or CTA.

In addition to rare anatomical variants, this patient also had a history of CABG using RGEA. As the population ages, we face patients with comorbidities more frequently. RGEA has been used for severe stenosis of the right coronary or left circumflex artery, a suitable conduit for CABG in terms of low surgical risk, high patency rate, and excellent patient outcome.^[23]^ Some case reports have documented surgical interventions in patients with gastric, hepatobiliary, and pancreatic malignancies who have previously received CABG via in situ RGEA graft.^[24,25]^ Surgery remains the only curative option in biliary and pancreatic malignancy. Patients with a CABG history must overcome the problem of cardiac perfusion. In other words, considering the resectability of the tumor during PD, GDA and RGEA must be preserved or reconstructed. There are 13 articles (including 5 Japanese articles)^[26–38]^ that discuss PD after CABG using RGEA (Table 2). There were 16 patients in total including our case, and one of them was female, with a mean age of 70 years. Ten patients (63%) underwent conventional PD, and three patients each underwent subtotal stomach preserving pancreaticoduodenectomy or pylorus-preserving pancreaticoduodenectomy. Due to tumor invasion or adequate lymph node dissection, RGEA grafts were sacrificed in 8 patients (50%). Preoperative revascularization was performed on 3 patients, percutaneous coronary intervention was performed on two patients, and preoperative additional bypass using saphenous vein graft from the axillary artery to RGEA was performed in 1 patient.^[38]^ In the abovementioned case studies, 6 patients needed intraoperative reconstruction of alternative bypass using GDA, CHA, splenic artery, or left gastric artery. Five out of the 6 patients utilized saphenous vein graft for the interposition of the vessels. All patients who sacrificed RGEA needed preoperative or intraoperative revascularization. Only one patient suffered from a pseudoaneurysm of RGEA and intraperitoneal bleeding. Transarterial coil embolization of the bypass graft was performed. There was no further adverse event, and no other complications or mortality were observed.

**Table 2:** Profiles of published cases underwent pancreaticoduodenectomy with coronary artery bypass grafting.

Year	Author	Age	Gender	Malignancy	Operation	Sacrificed RGEA	Revascularization	Cardiovascular event	Mortality
2019	Homsy et al^[26]^	73	M	Pancreatic cancer	PD	Yes	Intraoperative rerouting to GDA	None	None
2018	CreTu et al^[22]^	70	M	Pancreatic cancer	PD	Yes	Intraoperative rerouting to GDA interposition of SVG	None	None
		66	M	Pancreatic cancer	PD	Yes	Intraoperative rerouting to CHA interposition of SVG	None	None
		76	M	Bile duct cancer	PD	Yes	Intraoperative rerouting to LGA interposition of SVG	None	None
2015	Uemura et al^[23]^	73	M	Ampullary cancer	PPPD	No	None	Pseudoaneurysm of RGEA	None
2015	Ito et al^[24]^	63	M	Bile duct cancer	PD	No	Preoperative PCI to RCA	None	None
2014	Kitamura et al^[25]^	67	M	Bile duct cancer	SSPPD	No	None	None	None
2014	Fukuhara et al^[26]^	66	F	Ampullary cancer	PPPD	No	None	None	None
2014	Fujikawa et al^[27]^	64	M	Pancreatic cancer	SSPPD	Yes	Preoperative PCI to RCA	None	None
2013	Turcanu et al^[28]^	71	M	Pancreatic cancer	PD	No	None	None	None
2011	Takami et al^[29]^	80	M	Bile duct cancer	SSPPD	No	None	None	None
2011	Nakamura et al^[30]^	69	M	Ampullary cancer	PD	Yes	Intraoperative rerouting(graft unspecified)	None	None
2009	Kaji et al^[31]^	72	M	Pancreatic cancer	PPPD	No	None	None	None
2007	Mikawa et al^[32]^	72	M	Pancreatic cancer	PD	Yes	Intraoperative rerouting to SpAinterposition of SVG	None	None
2001	Ohtsuka et al^[33]^	62	M	Bile duct cancer	PD	Yes	Preoperative additional bypassusing SVG graft to AxA	None	None
2021	Our case	69	M	Ampullary cancer	PD	No	None	None	None

To our knowledge, this is the first reported case of a patient that underwent PD with a history of CABG utilizing RGEA graft and coexistence of rGDA. According to published cases and this case study, PD for such patients could be performed safely. To reduce the incidence of morbidity or mortality, preoperative assessment of vascular anomaly using MDCT is necessary. Furthermore, there is a need for combined vascular resection due to tumor invasion. Revascularization should be planned according to preoperative imaging examination.

## 4 Conclusion

Preoperative identification of vascular anomalies is the key to successful surgical intervention. MDCT and CTA revealed the anatomical variant precisely, and it contributed to intraoperative safety. The coexistence of CABG history made PD even more difficult for this patient. However, a CABG using RGEA in a patient with rare anatomical aberrant GDA should not be considered as an obstacle when R0 resection is achievable.

## Acknowledgments

We would like to thank Enago (http://www.enago.jp/) for the English language editing.

## Author contributions

**Data curation:** Satoshi Tokuda.

**Supervision:** Hideyuki Kanemoto, Akihiko Takagi, Noriyuki Oba.

**Writing – original draft:** Takeo Toda.

**Writing – review & editing:** Takeo Toda.
